# Associations of Hemostatic Variables with Cardiovascular Disease and Total Mortality: The Glasgow MONICA Study

**DOI:** 10.1055/a-1789-4896

**Published:** 2022-07-11

**Authors:** Gordon D. O. Lowe, Sanne A. E. Peters, Ann Rumley, Hugh Tunstall-Pedoe, Mark Woodward

**Affiliations:** 1 Institute of Cardiovascular and Medical Sciences, University of Glasgow, Glasgow, United Kingdom; 2 The George Institute for Global Health, University of New South Wales, Sydney, Australia; 3 Julius Center for Health Sciences and Primary Care, University Medical Center Utrecht, Utrecht University, Utrecht, The Netherlands; 4 The George Institute for Global Health, Imperial College London, London, United Kingdom; 5 Cardiovascular Epidemiology Unit, Institute of Cardiovascular Research, University of Dundee, Dundee, United Kingdom

**Keywords:** hemostasis, prevention, cardiovascular disease, mortality

## Abstract

The associations of plasma levels of hemostatic factors, other than fibrinogen, with risks of cardiovascular disease (CVD) and all-cause mortality are not well defined. In two phases of the Glasgow MONICA study, we assayed coagulation factors (VII, VIII, IX, and von Willebrand factor), coagulation inhibitors (antithrombin, protein C, protein S), coagulation activation markers (prothrombin fragment 1 + 2, thrombin–antithrombin complexes, D-dimer), and the fibrinolytic factors, tissue plasminogen activator (t-PA) antigen and plasminogen activator inhibitor type 1. Over 15 to 20 years, we followed up between 382 and 1,123 men and women aged 30 to 74 years, without baseline CVD, for risks of CVD and mortality. Age- and sex-adjusted hazard ratios (HRs) for CVD (top third vs bottom third) were significant only for factor VIII (1.30; 95% confidence interval [CI], 1.06–1.58) and factor IX (1.18; 95% CI, 1.01–1.39); these HRs were attenuated by further adjustment for CVD risk factors: 1.17 (95% CI, 0.94–1.46) and 1.07 (95% CI, 0.92–1.25), respectively. In contrast, factor VIII (HR, 1.63; 95% CI, 1.35–1.96), D-dimer (HR, 2.34; 95% CI, 1.26–4.35), and t-PA (HR, 2.81; 95% CI, 1.43–5.54) were strongly associated with mortality after full risk factor adjustment. Further studies, including meta-analyses, are required to assess the associations of these hemostatic factors with the risks of stroke and heart disease and causes of mortality.

## Introduction

Plasma levels of some hemostatic factors and activation markers have been associated with the risk of arterial cardiovascular diseases (CVDs): coronary heart disease (CHD), stroke, and peripheral arterial disease (PAD). 1 2 Fibrinogen is associated with risks of CHD, stroke, and total mortality 3 and may add to risk prediction scores for CVD risk. 4 5 A meta-analysis has established significant, but weaker, associations of plasma levels of von Willebrand factor (VWF), tissue plasminogen activator (t-PA) antigen, and the hemostatic activation marker fibrin D-dimer with the risk of CHD. 6 There is increasing evidence that these three markers are also associated with risks of stroke 7 and PAD. 8

For other hemostatic factors, inhibitors, and activation markers, there are insufficient data to assess with confidence their associations with CVD or mortality. 2 From the prospective Glasgow MONICA (Monitoring of Trends and Determinants of Cardiovascular Disease) study, we now report the associations with incident CVD (CHD and stroke), and with total mortality, of plasma levels of coagulation factors VII, VIII, and IX; coagulation activation markers prothrombin fragment 1 + 2 (F1 + 2), thrombin–antithrombin (TAT) complexes, and D-dimer; coagulation inhibitors antithrombin and protein C and protein S; activated partial thromboplastin time (aPTT), activated protein C (APC) resistance, and its common genetic determinant (the FV:R506Q mutation); and the fibrinolytic variables t-PA antigen and plasminogen activator inhibitor type 1 (PAI-1) activity.

## Methods

### Study Participants

The Glasgow MONICA study recruited random population samples of men and women aged 25 to 64 years in four phases: 1984–1987 (as part of the Scottish Heart Health Study); 1989; 1992 (aged 25–74 years); and 1995. 9 Data were subsequently combined to form the Scottish Heart Health Extended Cohort Study (SHHEC), from which a cardiovascular risk assessment score (the ASSIGN score) was developed for use in National Health Service Scotland. 10 All participants completed a questionnaire, which solicited information on demographics, past medical history, and lifestyle, including tobacco use. They were invited to attend clinics where blood pressure, weight, and height were measured, a 12-lead electrocardiogram (ECG) was applied, and a blood sample taken. The present study was restricted to those aged 30 to 74 years, free from clinical evidence of CVD at baseline, and having data on at least one hemostatic variable other than fibrinogen. CVD was diagnosed if they reported having received a previous doctor diagnosis of angina, heart attack, or stroke or previously undergoing coronary artery bypass graft (CABG) or percutaneous transluminal coronary angioplasty (PTCA) on the baseline questionnaire; if the ECG was suggestive of myocardial infarction (using Minnesota codes); or if the national records obtained showed that they had been hospitalized for CHD or stroke or had undergone any coronary surgical procedures prior to recruitment (and post-1980).

### Follow-up and End Point Definition

Individuals who gave written permission were followed up for cause-specific mortality through linkage with two national systems: death registrations and the record linkage database. 10 The latter was also used to follow up, and trace back, hospitalizations. Records were obtained from 1981 to 2009. 4 CVD end points were: deaths attributed to a cardiovascular cause (International Classification of Disease [ICD] 9 codes 390–459, ICD 10 codes 100–199); any hospital discharge diagnosis postrecruitment of CHD (ICD 9 codes 410–414, ICD 10 codes120–125) or cerebrovascular disease (ICD 9 codes 430–438, ICD 10 G45, 160–169); or surgical codes for CABG or PTCA.

### Hemostatic Variables

Blood was anticoagulated with 0.11 mol/L trisodium citrate (9:1 v/v) and centrifuged, and aliquots were stored at −80°C. Four hemostatic variables other than fibrinogen were studied in the 1989 survey. 11 VWF was assayed using an in-house enzyme-linked immunoassay (ELISA) employing rabbit antihuman polyclonal antibodies, obtained from DAKO plc, High Wycombe, United Kingdom. t-PA antigen was measured with a commercial ELISA (Biopool AB, Umea, Sweden). PAI-1 activity was measured with a commercial kit (Kabi, Sweden). D-dimer was assayed by a commercial ELISA (Dimertest; Agen Biomedical, Parsippany, New Jersey, United States). Significant baseline correlations with demographic and cardiovascular risk factor variables on multivariable analyses for these variables were age and triglycerides. 11

Ten other hemostatic variables were studied in the 1992 survey: coagulation factors VII, VIII, and IX, antithrombin and protein C and protein S activities, F1 + 2, TAT, aPTT, APC resistance, and the FV:R506Q mutation. Their assays, distributions, and correlations with baseline demographic and cardiovascular risk factor variables have been described. 12 13 14

### Statistical Methods

Data from the 1989 and 1992 MONICA cohorts were analyzed as two separate cohorts, reflecting the fact that different hemostatic variables were measured in the two baseline surveys. Cox proportional hazards models were used to estimate the hazard ratios (HRs) and 95% confidence intervals (CIs) for CVD, and total mortality, for each hemostatic variable. We predefined two models: model 1 was adjusted for age and sex only; model 2 was adjusted for the CVD risk factors also in the ASSIGN score: systolic blood pressure, serum total and high-density lipoprotein cholesterol, cigarettes smoked per day, diabetes status, family history of CHD, and the Scottish Index of Multiple Deprivation (SIMD) score. Analyses were conducted for a 1 standard deviation (SD) increase in level of each hemostatic variable; and for the highest third compared with the lowest third of its distribution. All statistical analyses were performed using R, version 2.15. 3 (R Foundation for Statistical Computing, Vienna, Austria).

## Results

### 1989 Survey: PAI-1, VWF, t-PA, D-dimer

Table 1 shows the baseline characteristics of participants in the second phase of the Glasgow MONICA study in 1989: baseline levels of PAI-1, VWF, D-dimer, and t-PA; and outcomes. During a median (interquartile interval) of 20.6 (20.5–20.8) years, 181 CVD events and 173 deaths occurred. Of 706 participants, citrated plasma aliquots were available, after routine measurement of plasma fibrinogen, from 523 participants for assay of PAI-1. Thereafter, as laboratory assays were developed sequentially for VWF, D-dimer, and t-PA antigens, residual plasma aliquots were sufficient for assay in 405, 384, and 382 participants, respectively.

**Table 1:** Baseline summary statistics: 1989 MONICA survey

Variable	*n*	
Cardiovascular risk factors		
Age (y), mean (SD)	706	48 (10)
Male sex, %	353	50
Systolic blood pressure (mm Hg), mean (SD)	706	132 (21)
Total cholesterol (mmol/L), mean (SD)	706	5.9 (1.2)
HDL cholesterol (mmol/L), mean (SD)	706	1.2 (0.3)
Body mass index (kg/m ^2^ ), mean (SD)	705	26 (4)
Current smokers, %	706	43
Diabetes, %	706	3
Family history of CVD, %	706	26
SIMD, median (IQI), mean (SD)	706	45 (24)
Hemostatic variables		
PAI-1 activity (AU/mL), mean (SD)	523	98.5 (46.5)
VWF (IU/dL), median (IQI)	405	91 (73–118)
D-dimer (ng/mL), median (IQI)	384	56 (37–78)
t-PA antigen (ng/mL), median (IQI)	382	3.9 (5.5–8.5)

Abbreviations: CVD, cardiovascular disease; HDL, high-density lipoprotein; IQI, interquartile interval; PAI-1, plasminogen activator inhibitor type 1; SD, standard deviation; t-PA, tissue plasminogen activator; VWF, von Willebrand factor.

Note: Decreasing number of available samples reflects decreasing availability of plasma aliquot.

Table 2 shows the associations of these hemostatic variables with the study outcomes, both as a 1 SD increase in levels and as ratio of top third to bottom third of levels. PAI-1 and VWF showed no association with CVD or mortality after full adjustment for ASSIGN variables. D-dimer and t-PA showed associations with CVD and mortality after full adjustment—HRs, top third versus bottom third: 2.34 (95% CI, 1.26–4.35) and 2.81 (95% CI, 1.43–5.54); and per 1 SD increase: 1.16 (95% CI, 0.99–1.36) and 1.63 (95% CI, 1.35–1.96).

**Table 2:** Associations with outcomes: 1989 MONICA survey

	CVD		Total mortality	
	Adjustments		Adjustments	
Variable	Age and sex	ASSIGN	Age and sex	ASSIGN
HRs for a 1 SD increase in level				
PAI-1	1.18 (1.01–1.39)	1.07 (0.92–1.25)	1.12 (0.95–1.32)	1.06 (0.90–1.25)
VWF	1.02 (0.85–1.23)	0.99 (0.83–1.18)	1.05 (0.88–1.25)	1.01 (0.84–1.20)
D-dimer	1.18 (1.01–1.37)	1.17 (1.00–1.38)	1.18 (1.02–1.38)	1.16 (0.99–1.36)
t-PA	1.30 (1.06–1.58)	1.17 (0.94–1.46)	1.62 (1.36–1.92)	1.63 (1.35–1.96)
HRs for top third vs bottom third of distribution				
PAI-1	1.08 (0.72–1.62)	0.95 (0.63–1.44)	1.20 (0.78–1.86)	1.11 (0.71–1.74)
VWF	1.01 (0.62–1.65)	1.03 (0.63–1.69)	1.10 (0.67–1.80)	1.01 (0.61–1.66)
D-dimer	1.28 (0.77–2.13)	1.39 (0.83–2.32)	2.26 (1.23–4.15)	2.34 (1.26–4.35)
t-PA	1.49 (0.84–2.63)	1.09 (0.61–1.97)	3.20 (1.64–6.24)	2.81 (1.43–5.54)

Abbreviations: CVD, cardiovascular disease; HRs, hazard ratios; PAI-1, plasminogen activator inhibitor type 1; SD, standard deviation; t-PA, tissue plasminogen activator; VWF, von Willebrand factor.

Note: HRs and 95% confidence intervals for CVD and total mortality. Analyzed first for a 1 SD increase in hemostatic factor level and then for top third versus bottom third of distribution of level. Adjusted for age and sex and then also for ASSIGN risk variables (systolic blood pressure, serum total and high-density lipoprotein cholesterol, cigarettes smoked per day, diabetes status, family history of coronary heart disease, and the Scottish Index of Multiple Deprivation [SIMD] score).

### 1992 Survey: Factors VII, VIII, and IX, Antithrombin, Protein C, Protein S, F1 + 2, TAT, aPTT, APC Resistance, FV:R506Q Mutation

Table 3 shows the baseline characteristics of participants in the third phase of the Glasgow MONICA study in 1992; baseline levels of coagulation factors, inhibitors, and activation markers; and outcomes. During a median (interquartile interval) of 17.5 (15.4–17.8) years, 381 CVD events and 401 deaths occurred. Of 1,324 participants, citrated plasma aliquots were available, after routine measurement of plasma fibrinogen, from 1,088 to 1,123 participants for assay of factors VII, VIII, IX, antithrombin, and protein C activities. Thereafter, as laboratory assays were developed sequentially for protein S, F1 + 2, and TAT antigens, residual plasma aliquots were sufficient for assay in 843 to 896 participants and then for aPTT and APC ratio in 709 to 715 participants.

**Table 3:** Baseline summary statistics: 1992 MONICA survey

Variable	*n*	
Cardiovascular risk factors		
Age (y), mean (SD)	1,324	51 (13)
Male sex, %	1,324	47
Systolic blood pressure (mm Hg), mean (SD)	1,324	133 (24)
Total cholesterol (mmol/L), mean (SD)	1,324	6.1 (1.2)
HDL cholesterol (mmol/L), mean (SD)	1,324	1.5 (0.4)
Body mass index (kg/m ^2^ ), mean (SD)	1,313	26 (5)
Current smokers, %	1,324	42
Diabetes, %	1,324	1
Family history of CVD, %	1,324	28
SIMD, median (IQI), mean (SD)	1,324	46 (24)
Hemostatic variables		
Factor VII (IU/dL), mean (SD)	1,115	109.9 (24.3)
Factor VIII (IU/dL), mean (SD)	1,121	146.3 (50.0)
Factor IX (IU/dL), mean (SD)	1,113	131.4 (33.8)
Antithrombin (IU/dL), mean (SD)	1,088	100.2 (12.9)
Protein C (IU/dL), mean (SD)	1,123	108.6 (31.9)
Protein S (%pool), mean (SD)	896	130.7 (29.4)
F1 + 2 (ng/mL), median (IQI)	887	2.12 (1.53–2.95)
TAT (ng/mL), median (IQI)	843	4.09 (2.73–7.36)
aPTT (s), mean (SD)	709	31.1 (4.3)
APC ratio, mean (SD)	715	2.86 (0.54)
Heterozygous FV:R506Q (%)	1293	2.5

Abbreviations: APC, activated protein C; aPTT, activated partial thromboplastin time; CVD, cardiovascular disease; F1 + 2, prothrombin fragment 1 + 2; HDL, high-density lipoprotein; IQI, interquartile interval; SD, standard deviation; TAT, thrombin–antithrombin.

Note: Decreasing number of available samples reflects decreasing availability of plasma aliquots.

Table 4 shows the associations of these hemostatic variables with the study outcomes, both as a 1 SD increase in levels and as ratio of top third to bottom third of levels. Age- and sex-adjusted HRs for CVD, top third versus bottom third, were significant only for factor VIII (1.30; 95% CI, 1.06–1.58) and factor IX (1.18; 95% CI, 1.01–1.39); these HRs were attenuated by further adjustment for CVD risk factors, respectively, to 1.17 (95% CI, 0.94–1.46) and 1.07 (95% CI, 0.92–1.25). In contrast, factor VIII was strongly associated with total mortality after full risk factor adjustment: HRs, top third versus bottom third, 1.63 (95% CI, 1.35–1.96) and per 1 SD increase 1.14 (95% CI, 1.03–1.28). No other hemostatic variable measured in this survey was significantly associated with either CVD or with total mortality.

**Table 4:** Associations with outcomes: 1992 MONICA survey

	CVD		Total mortality	
	Adjustments		Adjustments	
	Age and sex	ASSIGN score	Age and sex	ASSIGN score
HRs for a 1 SD increase in level (* log transformed)				
Factor VII	1.12 (1.00–1.26)	1.07 (0.95–1.21)	1.09 (0.97–1.22)	1.09 (0.97–1.23)
Factor VIII	1.10 (0.99–1.22)	1.09 (0.97–1.22)	1.13 (1.02–1.26)	1.14 (1.03–1.28)
Factor IX	1.10 (0.98–1.22)	1.02 (0.91–1.15)	1.09 (0.98–1.21)	1.06 (0.94–1.18)
Antithrombin	1.06 (0.95–1.18)	1.10 (0.89–1.12)	1.02 (0.92–1.14)	1.00 (0.89–1.11)
Protein C	0.94 (0.84–1.05)	0.92 (0.82–1.04)	0.90 (0.81–1.01)	0.91 (0.81–1.02)
Protein S	1.14 (1.00–1.30)	1.06 (0.93–1.20)	1.08 (0.95–1.23)	1.03 (0.90–1.17)
F1 + 2*	1.12 (0.99–1.27)	1.09 (0.96–1.25)	1.02 (0.90–1.15)	1.02 (0.90–1.16)
TAT*	1.04 (0.91–1.19)	1.02 (0.89–1.17)	0.96 (0.85–1.09)	0.94 (0.82–1.07)
aPTT	0.94 (0.81–1.09)	0.92 (0.79–1.08)	1.03 (0.90–1.18)	1.03 (0.89–1.19)
APC ratio	0.97 (0.83–1.13)	1.00 (0.86–1.16)	0.98 (0.84–1,14)	0.98 (0.84–1.14)
HRs for top third versus bottom third of distribution				
Factor VII	1.02 (0.85–1.23)	0.99 (0.83–1.18)	1.05 (0.88–1.25)	1.01 (0.84–1.20)
Factor VIII	1.30 (1.06–1.58)	1.17 (0.94–1.46)	1.62 (1.36–1.92)	1.63 (1.35–1.96)
Factor IX	1.18 (1.01–1.39)	1.07 (0.92–1.25)	1.12 (0.95–1.32)	1.06 (0.90–1.25)
Antithrombin	1.25 (0.95–1.64)	1.11 (0.84–1.47)	1.19 (0.91–1.55)	1.12 (0.85–1.48)
Protein C	0.84 (0.64–1.10)	0.82 (0.62–1.08)	0.82 (0.63–1.06)	0.84 (0.64–1.10)
Protein S	1.18 (0.86–1.62)	1.03 (0.75–1.41)	1.17 (0.85–1.60)	1.04 (0.75–1.43)
F1 + 2	1.37 (0.99–1.89)	1.23 (0.89–1.71)	1.15 (0.84–1.58)	1.11 (0.81–1.52)
TAT	0.96 (0.69–1.33)	0.94 (0.68–1.31)	0.86 (0.63–1.18)	0.81 (0.59–1.12)
aPTT	1.06 (0.74–1.52)	0.98 (0.68–1.41)	1.17 (0.82–1.66)	1.16 (0.81–1.66)
APC ratio	0.94 (0.65–1.35)	1.03 (0.70–1.49)	0.95 (0.67–1,35)	0.99 (0.69–1.42)

Abbreviations: APC, activated protein C; aPTT, activated partial thromboplastin time; CVD, cardiovascular disease; F1 + 2, prothrombin fragment 1 + 2; HRs, hazard ratios; SD, standard deviation; TAT, thrombin–antithrombin.

Note: HRs and 95% confidence intervals for CVD and total mortality. Analyzed first for a 1 SD increase in hemostatic factor level and then for top third versus bottom third of distribution of level. Adjusted for age and sex and then also for ASSIGN risk variables (systolic blood pressure, serum total and high-density lipoprotein cholesterol, cigarettes smoked per day, diabetes status, family history of coronary heart disease, and the Scottish Index of Multiple Deprivation [SIMD] score). Log transformation was required for variable F1+2 and TAT, for calculation of HRs for a 1SD increase in level (top half of Table 4 ).

## Discussion

In this study of the associations of plasma levels of 15 hemostatic variables with risks of CVD, and of total mortality, in two random population samples of men and women aged 30 to 74 years, age- and sex-adjusted HRs for CVD were significant for factor VIII, factor IX, D-dimer, and t-PA. Apart from D-dimer, these were attenuated by further adjustment for CVD risk factors. In contrast, factor VIII, D-dimer, and t-PA were strongly associated with total mortality after full risk factor adjustment.

Factor VII level was not associated with CVD or mortality in our study after risk factor adjustment. This is consistent with recent reviews of studies 2 15 and with meta-analyses of functional mutations in the factor VII gene, which showed no associations with CHD. 15 16

The association of factor VIII level with CVD (HR, top to bottom third, 1.30; 95% CI, 1.06–1.58) was attenuated after risk factor adjustment. There is uncertainty in the literature on this association. 2 17 18 19 20 21 22 23 24 25 26 We observed no significant association of its carrier protein, VWF, with CVD; a meta-analysis of the association of VWF level with CHD in 15 studies with 6,556 cases showed a significant association: HR per 1 SD, 1.16 (95% CI, 1.10–1.22). 6 Plasma VWF, as its carrier protein, is correlated with plasma factor VIII. 17 18

Plasma factor VIII was associated with total mortality in our study, after risk factor adjustment (HR, 1.63; 95% CI, 1.35–1.96). This association has also been reported from the Multi-Ethnic Study of Atherosclerosis 19 and the MEGA study, 20 which suggested that environmental factors, such as chronic comorbidities and chronic inflammation, are at least in part responsible. While we did not observe an association of VWF with mortality in the present study, this association was found in the MEGA study. 20

Factor IX level was weakly associated with CVD after adjustment for age and sex (HR, top to bottom third, 1.18; 95% CI, 1.01–1.39) and attenuated after further adjustment for risk factors in the ASSIGN score; (HR, 1.07; 95% CI, 0.92–1.25). As with factor VIII, there is uncertainty in the literature on this association. 2 27 28 We did not observe an association of factor IX with mortality.

Among coagulation activation markers, F1 + 2 showed a borderline association with CHD when adjusted for age and sex, but no coagulation activation marker was associated with incident CVD after additional adjustment for risk factors. Studies of the associations of F1 + 2 and TAT complexes with risk of CVD are inconclusive. 21 22 23 A meta-analysis found a significant association of D-dimer levels with CHD: HR, 1.23 (95% CI, 1.16–1.32; 18 studies with 6,799 cases). 6

Among coagulation inhibitors, protein S showed a borderline association with CHD when adjusted for age and sex; but no coagulation inhibitor was associated with incident CVD after additional adjustment for age, sex, and risk factors in the ASSIGN score nor with total mortality. The Atherosclerosis Risk in Communities (ARIC) study found a similar lack of association for antithrombin and protein C for CHD 24 25 but found an increased risk of stroke for low protein C in longer-term follow-up. 25 26 A meta-analysis confirmed increased risk of stroke for deficiencies of proteins C and S, but not of antithrombin. 29 No increase in mortality has been reported in persons with deficiencies of coagulation inhibitors. 30

aPTT was not associated with CHD in our study, as in the ARIC study. 26 27 Neither APC resistance nor its major genetic determinant, the FV:R506Q mutation, was associated with CHD in our study; however, meta-analyses have shown significant associations of FV:R506Q with CHD 16 and stroke. 29 We observed a borderline association of FV:R506Q with total mortality, but this was not observed in a larger study. 30

The fibrinolytic variables, tPA antigen and PAI-1 activity, did not show significant adjusted associations with incident CVD in our study. The association of tPA was, however, consistent with a meta-analysis of incident CHD (HR, 1.13; 95% CI, 1.06–1.21; 13 studies with 5,494 cases). 6 The association of PAI-1 with incident CVD was consistent with that in another meta-analysis of its associations with CHD risk, which reported an HR for top third to bottom third of 0.98 (95% CI, 0.53–1.87). 31

In contrast, t-PA was significantly associated with total mortality after adjustment for age, sex, and risk factors in the ASSIGN score (HR, top third to bottom third, 2.81; 95% CI, 1.43–5.54). This association was also observed in the PROSPER study cohort. 23

Strengths of our study include that it is a large random sample of a general population of men and women aged 30 to 74 years, followed for up to 20 years for both CVD and total mortality. Also, the study assessed 15 hemostatic variables in a combined analysis, using well-calibrated laboratory assays, applying national or international standards, where available. 12 We had a limited number of outcome events, which precluded reliable estimation of associations with stroke and CHD separately or of causes of mortality. We were also unable to look at changes in hemostatic variables over time, having only baseline values. Another limitation was the largely Scottish ethnic population, and hence results may not be generalizable to other populations, worldwide.

## Conclusion

In this study, we found no compelling evidence of independent associations of hemostatic variables with CVD, suggesting that they may not add significantly to CVD risk stratification. We report that factor VIII, D-dimer, and t-PA were strongly associated with total mortality after full risk factor adjustment, but found no such association for other hemostatic variables. Further studies and meta-analyses are required to reliably assess the associations of the hemostatic variables we have studied with the risks of components of CVD, particularly stroke and CHD, 6 and causes of mortality.

## Notes

**What Is Known about This Topic?**

- Apart from fibrinogen, there are limited data on the associations of hemostatic factors with risks of CVD and total mortality.

**What Does This Paper Add?**

- Of 15 hemostatic variables assayed in a well-defined cohort followed for 17 years, factors VIII and IX were associated with risks of CVD after adjustment for age and sex; but these associations were attenuated after adjustment for CVD risk factors.
- Factor VIII, D-dimer, and t-PA were associated with total mortality after adjustment for age, sex, and CVD risk factors.

## References

[1] Lowe G DO Can haematological tests predict cardiovascular risk? The 2005 Kettle Lecture Br J Haematol 2006 133 03 232 250 16643425 10.1111/j.1365-2141.2006.06021.x

[2] Lowe G Rumley A The relevance of coagulation in cardiovascular disease: what do the biomarkers tell us? Thromb Haemost 2014 112 05 860 867 25231258 10.1160/TH14-03-0199

[3] Fibrinogen Studies Collaboration Danesh J Lewington S Thompson S G Plasma fibrinogen level and the risk of major cardiovascular diseases and nonvascular mortality: an individual participant meta-analysis JAMA 2005 294 14 1799 1809 16219884 10.1001/jama.294.14.1799

[4] Woodward M Tunstall-Pedoe H Rumley A Lowe G DO Does fibrinogen add to prediction of cardiovascular disease? Results from the Scottish Heart Health Extended Cohort Study Br J Haematol 2009 146 04 442 446 19549268 10.1111/j.1365-2141.2009.07778.x

[5] Emerging Risk Factors Collaboration Kaptoge S Di Angelantonio E Pennells L C-reactive protein, fibrinogen, and cardiovascular disease prediction N Engl J Med 2012 367 14 1310 1320 23034020 10.1056/NEJMoa1107477 PMC3714101

[6] Willeit P Thompson A Aspelund T Hemostatic factors and risk of coronary heart disease in general populations: new prospective study and updated meta-analyses PLoS One 2013 8 02 e55175 23408959 10.1371/journal.pone.0055175 PMC3567058

[7] Wannamethee S G Whincup P H Lennon L Rumley A Lowe G D Fibrin D-dimer, tissue-type plasminogen activator, von Willebrand factor, and risk of incident stroke in older men Stroke 2012 43 05 1206 1211 22382157 10.1161/STROKEAHA.111.636373

[8] Tzoulaki I Murray G D Lee A J Rumley A Lowe G DO Fowkes F GR Inflammatory, haemostatic, and rheological markers for incident peripheral arterial disease: Edinburgh Artery Study Eur Heart J 2007 28 03 354 362 17213229 10.1093/eurheartj/ehl441

[9] Tunstall-Pedoe H Kuulasmaa K Tolonen H MONICA Monograph and Multimedia Sourcebook: World's Largest Study of Heart Disease, Stroke, Risk Factors, and Population Trends 1979–2002 Geneva World Health Organization 2003

[10] SIGN group on risk estimation Woodward M Brindle P Tunstall-Pedoe H Adding social deprivation and family history to cardiovascular risk assessment: the ASSIGN score from the Scottish Heart Health Extended Cohort (SHHEC) Heart 2007 93 02 172 176 17090561 10.1136/hrt.2006.108167 PMC1861393

[11] Rumley A Fibrinolytic and Endothelial Markers in Cardiovascular Disease and Diabetes Mellitus [PhD Thesis] Glasgow University of Glasgow 1996

[12] Lowe G DO Rumley A Woodward M Epidemiology of coagulation factors, inhibitors and activation markers: the Third Glasgow MONICA Survey. I. Illustrative reference ranges by age, sex and hormone use Br J Haematol 1997 97 04 775 784 9217176 10.1046/j.1365-2141.1997.1222936.x

[13] Woodward M Lowe G DO Rumley A Epidemiology of coagulation factors, inhibitors and activation markers: The Third Glasgow MONICA Survey. II. Relationships to cardiovascular risk factors and prevalent cardiovascular disease Br J Haematol 1997 97 04 785 797 9217177 10.1046/j.1365-2141.1997.1232935.x

[14] Lowe G DO Rumley A Woodward M Reid E Rumley J Activated protein C resistance and the FV:R506Q mutation in a random population sample–associations with cardiovascular risk factors and coagulation variables Thromb Haemost 1999 81 06 918 924 10404768

[15] Bernardi F Mariani G Biochemical, molecular and clinical aspects of coagulation factor VII and its role in hemostasis and thrombosis Haematologica 2021 106 02 351 362 33406812 10.3324/haematol.2020.248542 PMC7849579

[16] Ye Z Liu E HC Higgins J PT Seven haemostatic gene polymorphisms in coronary disease: meta-analysis of 66,155 cases and 91,307 controls Lancet 2006 367(9511):651 658 16503463 10.1016/S0140-6736(06)68263-9

[17] Rumley A Lowe G DO Sweetnam P M Yarnell J WG Ford R P Factor VIII, von Willebrand factor and the risk of major ischaemic heart disease in the Caerphilly Heart Study Br J Haematol 1999 105 01 110 116 10233372

[18] Rietveld I M Lijfering W M le Cessie S High levels of coagulation factors and venous thrombosis risk: strongest association for factor VIII and von Willebrand factor J Thromb Haemost 2019 17 01 99 109 30471183 10.1111/jth.14343

[19] Multiethnic Study of Atherosclerosis Investigators Folsom A R Delaney J AC Lutsey P L Associations of factor VIIIc, D-dimer, and plasmin-antiplasmin with incident cardiovascular disease and all-cause mortality Am J Hematol 2009 84 06 349 353 19472201 10.1002/ajh.21429 PMC2950108

[20] Yap E S Timp J F Flinterman L E Elevated levels of factor VIII and subsequent risk of all-cause mortality: results from the MEGA follow-up study J Thromb Haemost 2015 13 10 1833 1842 26264493 10.1111/jth.13071

[21] Cooper J A Miller G J Bauer K A Comparison of novel hemostatic factors and conventional risk factors for prediction of coronary heart disease Circulation 2000 102 23 2816 2822 11104738 10.1161/01.cir.102.23.2816

[22] Smith A Patterson C Yarnell J Rumley A Ben-Shlomo Y Lowe G Which hemostatic markers add to the predictive value of conventional risk factors for coronary heart disease and ischemic stroke? The Caerphilly Study Circulation 2005 112 20 3080 3087 16286603 10.1161/CIRCULATIONAHA.105.557132

[23] Rumley A Lowe G Stott D Coagulation activation markers are associated with cardiovascular events and death in the elderly at risk: PROSPER study cohortJ Thromb Haemost 2009;7(Suppl 2):Abstract OC-WE-33

[24] Folsom A R Wu K K Rosamond W D Sharrett A R Chambless L E Prospective study of hemostatic factors and incidence of coronary heart disease: the Atherosclerosis Risk in Communities (ARIC) Study Circulation 1997 96 04 1102 1108 9286936 10.1161/01.cir.96.4.1102

[25] Folsom A R Ohira T Yamagishi K Cushman M Low protein C and incidence of ischemic stroke and coronary heart disease: the Atherosclerosis Risk in Communities (ARIC) Study J Thromb Haemost 2009 7 11 1774 1778 19691480 10.1111/j.1538-7836.2009.03577.x PMC2819378

[26] The Atherosclerosis Risk in Communities (ARIC) Study Investigators Folsom A R Rosamond W D Shahar E Prospective study of markers of hemostatic function with risk of ischemic stroke Circulation 1999 100 07 736 742 10449696 10.1161/01.cir.100.7.736

[27] ARIC Study Inverstigators Yamagishi K Aleksic N Hannan P J Folsom A R Coagulation factors II, V, IX, X, XI, and XII, plasminogen, and alpha-2 antiplasmin and risk of coronary heart disease J Atheroscler Thromb 2010 17 04 402 409 20379055 10.5551/jat.3673 PMC2866762

[28] Olson N C Cushman M Judd S E Associations of coagulation factors IX and XI levels with incident coronary heart disease and ischemic stroke: the REGARDS study J Thromb Haemost 2017 15 06 1086 1094 28393470 10.1111/jth.13698 PMC9797027

[29] Chiasakul T De Jesus E Tong J Inherited thrombophilia and the risk of arterial ischemic stroke: a systematic review and meta-analysis J Am Heart Assoc 2019 8 19 e012877 31549567 10.1161/JAHA.119.012877 PMC6806047

[30] Pabinger I Vossen C Y Lang J Mortality and inherited thrombophilia: results from the European Prospective Cohort on Thrombophilia J Thromb Haemost 2012 10 02 217 222 22128841 10.1111/j.1538-7836.2011.04573.x

[31] Lowe G DO Danesh J Lewington S Tissue plasminogen activator antigen and coronary heart disease. Prospective study and meta-analysis Eur Heart J 2004 25 03 252 259 14972427 10.1016/j.ehj.2003.11.004

